# Back and neck pain are related to mental health problems in adolescence

**DOI:** 10.1186/1471-2458-11-382

**Published:** 2011-05-25

**Authors:** Clare S Rees, Anne J Smith, Peter B O'Sullivan, Garth E Kendall, Leon M Straker

**Affiliations:** 1 School of Psychology and Speech Pathology, Curtin University, GPO Box U1987, Perth, WA 6845, Australia; 2 School of Physiotherapy, Curtin University, GPO Box U1987, Perth, WA 6845, Australia; 3 School of Nursing and Midwifery, Curtin University, GPO Box U1987, Perth, WA 6845, Australia; 4 Telethon Institute for Child Health Research, Roberts Road, Perth, WA 6008, Australia; 5 Curtin Health Innovation Research Institute, Curtin University, GPO Box U1987, Perth, WA 6845, Australia

**Keywords:** Child Behaviour Check List, Adolescence, Spinal Pain, Mental Health, Raine Study

## Abstract

**Background:**

There is a high prevalence of mental health problems amongst adolescents. In addition there is a high prevalence of spinal pain in this population. Evidence suggests that these conditions are related. This study sought to extend earlier findings by examining the relationship between mental health problems as measured by the Child Behaviour Check List (CBCL) and the experience of back and neck pain in adolescents.

**Methods:**

One thousand five hundred and eighty participants (mean age 14.1 years) from the Western Australian Pregnancy (Raine) Study provided cross-sectional spinal pain and CBCL data.

**Results:**

As predicted, there was a high prevalence of back and neck pain in this cohort. On the whole, females reported more mental health difficulties than males. There were strong relationships between the majority of symptom scales of the CBCL and back and neck pain. Scores on the CBCL were associated with higher odds of comorbid back and neck pain.

**Conclusions:**

These findings strongly support the need to consider both psychological and pain symptoms when providing assessments and treatment for adolescents. Further research is required to inform causal models.

## Background

Significant biological, emotional, intellectual and social changes take place during adolescence and mental health problems are relatively common in this age group. The population point- prevalence of mental health disorder in those aged between 13 and 17 years residing in Australia and the United States has been reliably estimated at around 11% - 12% [1,2]. Adolescent mental health problems may be short-term states, or persistent long-term patterns [3]. Persistent long-term patterns are of concern as they may be precursors of adult psychopathology. Many of the common forms of child and adolescent mental health problems are substantially correlated [4]. Notwithstanding this, many adult disorders, such as major depression and social phobia, definitely occur in childhood and adolescence, starting much earlier than used to be thought possible [5]. For example, major depression is a common and serious disorder of adolescence. Lifetime prevalence increases dramatically from 1% of the population under 12 to approximately 17% - 25% by the end of adolescence [6]. Genetic factors and exposure to early adversity in the form of socioeconomic disadvantage, parental mental illness or substance use, family dysfunction, and stressful life-events are key risk factors [6,7]. The 2:1 female: male prevalence ratio for adolescent unipolar depression is a well established finding, suggesting that some aspect of the female hypothalamic-pituitary-gonadal system may be responsible for inducing and maintaining a substantial proportion of these states [8]. The experience of current depression plus anxiety greatly increases risk for suicidality [9].

The experience of physical pain is also a common problem in this age group. A study of 5424 children and adolescents by Perquin and colleagues [10] found that over 50% had experienced pain in the last 3 months and that for a quarter of the sample the pain was chronic. The most common types of pain reported by adolescents are headaches, stomach, back, and limb/musculoskeletal pain with much comorbidity [11]. Low back pain is very common in adolescence (up to 46% by the age of 14 years) [12] and an organic cause is rarely found. The presence of this disorder in adolescence increases its risk for chronicity in adulthood [13]. In children and adolescents, neck and shoulder pain is also common [14] and there is significant co-morbidity with back pain, especially in adolescents [15,16]. As with mental health problems, sex differences in reports of pain become apparent around puberty [17]. The study by Perquin and colleagues [10] found that girls tended to report more chronic and severe pain than boys, particularly girls aged between 12 and 14 years. A study by Roth-Isigkeit, Thyen, Raspe, Stoven, and Schmucker [18] found the 3-month prevalence of any pain amongst adolescents aged 13-15 years or 16-18 years was significantly higher for girls than for boys. Sundblad, Saartok and Engstrom [19] conducted a study of Swedish students and examined the seven day prevalence of headache, abdominal and musculoskeletal pain. The authors found no difference in the prevalence of musculoskeletal pain between boys and girls but found that girls were twice as likely to report headache and abdominal pain. An earlier study by Mikkelsson, Salminen and Kautiainen [20] of children in Finland also found mixed results with girls reporting more chest and upper back pain than boys whereas no differences were found for pain in the lower back or neck.

Given the substantial rates of mental health problems and the common experience of pain in this age group, and the possibility of a shared neurobiological pathway for these disorders [21], the relationship between them has already been the subject of considerable research. A study by Larsson and Sund [22] found that pain frequency was strongly related to levels of both internalising and externalising problems among adolescents irrespective of pain location. There are also sex differences in the experience of pain and associated mental health problems. In epidemiological studies, depressive and anxiety disorders have been associated with recurrent headaches and stomach aches in girls but not boys, while musculoskeletal pain has been related to depression in both sexes [23].

In a study of 1446 children aged 11-14 years, Watson and colleagues [24] found that low back pain was strongly associated with emotional problems, conduct problems and general somatic complaints. In this study children completed the Strengths and Difficulties Questionnaire (SDQ) which is a brief behavioural screening tool consisting of 25 items [25]. Four, 5-item scales make up the 'Difficulties' score (emotional symptoms, conduct problems, hyperactivity, peer relationships) and a 5-item prosocial scale representing 'Strengths'. The authors also measured somatic complaints by asking the children how many times in the past month they had experienced headaches, abdominal pain, and sore throats. This important study highlights the strong relationship between low back pain and mental health problems albeit with a somewhat restricted measure of psychological functioning. Although the SDQ has good validity, the limited number of items restricts the level of detail that can be extrapolated as to overall levels of psychological functioning. The Youth Self Report (YSR) scale of the Child Behaviour Check List (CBCL) is a widely used measure that correlates highly with the SDQ. It is a more comprehensive measure of adolescent mental health as it includes emotional and behavioural problems consisting of 118 items producing 8 syndrome scales. One aim of the present study is to utilise the CBCL in order to gain a more comprehensive measure of the mental health of adolescents with spinal pain.

It is surprising that neck pain has not received the same amount of research attention as low back pain, given its high prevalence in the general population and in adolescent cohorts [26]. Hogg-Johnston and colleagues [27] conducted a best evidence synthesis of the burden and prevalence of neck pain and reported studies showing that poor psychological health both predicts and coexists with the experience of neck pain. In addition to this there is evidence that neck pain is specifically associated with depression [28]. In one of the rare child studies including neck pain, Murphy and colleagues [14] studied 679 school children (aged 11-14 years) in the United Kingdom. They investigated the relationship between low back pain, upper back pain, neck pain and psychological functioning, using the SDQ. They found that all three types of spinal pain were associated with mental health problems as well as the reporting of other somatic complaints, in this case, sore throat and headaches. Although this study included neck pain, like the Watson study it used the SDQ thus limiting the breadth of information garnered regarding the relationship between different types of pain and mental health problems.

The aim of this study is to investigate the relationship between spinal pain and mental health problems in a large cohort of Australian adolescents. In particular, this study will extend the findings of earlier studies by utilising the CBCL which is a more comprehensive measure of mental health problems. The study will also investigate the relationship between adolescent neck pain and psychological functioning which has until now been largely neglected in the literature. A further aim is to examine sex differences in pain experience and related mental health.

### Hypotheses

Higher levels of mental health problems (as measured by internalising, externalising and other symptoms) in adolescence, as measured by the CBCL, will be associated with increased odds of the experience of either back or neck pain, and further increased odds of co-morbid back and neck pain.

Girls will report more mental health problems than boys, as measured by the CBCL, but associations between mental health problems and the experience of back and/or neck pain will be of similar strength across sexes.

## Methods

### Participants

Participants for this cross-sectional study were adolescents enrolled in the West Australian Pregnancy Cohort "Raine" Study [12,29]. The Raine study started as a pregnancy cohort of women enrolled at or before the 18th week of gestation from the public antenatal clinic at the principal obstetric hospital in Perth, Western Australia, or nearby private practices. Women were enrolled from August 1989 to April 1992. Comparative analysis at 14 years showed that the cohort remained representative of the Western Australian population. One thousand five hundred and eighty participants (mean age 14.1 years, standard deviation 0.2) provided spinal pain and CBCL data at the 14 year follow-up from a total of 1608 adolescents participating. Informed consent was obtained from parents or guardians. The study was approved by the Ethics and Scientific Review Committee of Princess Margaret Hospital for Children and the Human Research Ethics Committee of Curtin University.

### Questionnaire

As part of a larger study participants completed a questionnaire covering a range of physical, psychosocial and medical issues on a laptop computer. For this study the subjects were specifically asked; (i) Have you ever had back pain?, and if they reported pain ever, (ii) Has your back been painful in the last month? Adolescents were also asked about their experience of neck/shoulder pain, described as pain in the area of the posterior neck and upper trapezius; (i) Have you ever had neck/shoulder pain?, and if they reported pain ever, (ii) Has your neck/shoulder been painful in the last month? These questions are based on the Nordic pain questionnaire, and have established validity and reliability for assessing spinal pain [30-35].

A composite dependent variable was derived from responses to the questions regarding experience of back and neck/shoulder pain in the last month. Categories were i) No back or neck/shoulder pain in the last month, ii) Neck/shoulder pain only in the last month iii) Back pain only in the last month iv) Neck/shoulder pain and back pain in the last month.

Adolescents also completed the YSR of the CBCL [36]. The reliability and validity of the CBCL has been extensively examined [3], and the utility has been documented in Australian settings [37,38]. The 118 items of the YSR form eight syndrome scales; Somatic Complaints, Anxious/Depressed, Withdrawn, Aggressive Behaviour, Rule-breaking Behaviour, Social Problems, Thought Problems and Attention Problems. Prior second order factor analysis identified two broad-band scales; Internalising (reflecting problems of Anxiety/Depression, Withdrawal and Somatic Complaints scales), and Externalising (reflecting Rule-breaking and Aggressive Behaviour). Raw scores for the eight YSR syndrome scales were used for analysis as recommended by Achenbach [39] for research involving distinctions between children with mild symptoms.

#### Statistical Analysis

Statistical analysis was performed using Stata/IC 10.1 for Windows (Statacorp LP, College Station TX). Chi-squared tests or two-sample t-tests were used to evaluate sex differences in pain prevalence and YSR syndrome scale scores. Eight multinomial logistic regression models (one for each scale of the YSR) were used to examine the association between the four category dependent variable of spinal pain and the YSR syndrome scores, adjusting for the confounding effect of sex. The potential for differences in effect between sexes was determined by evaluation of a sex interaction term. In the presence of a significant sex interaction term, separate effect estimates for males and females are provided. Statistical significance for main effects was set at α = 0.05 and for interaction effects at α = 0.10. Linearity of effects was confirmed by examination of plots of pain prevalences by deciles of YSR scores. Risk ratios are displayed as the ratio of risk for a change in the interquartile range of the score, i.e. moving from 25^th ^to 75^th ^percentile, so that effect sizes are comparable across the differently scales.

## Results

### Pain prevalence

Table 1 displays the prevalence of back and/or neck pain in the last month overall and by sex. Females had significantly higher prevalence of neck pain only, and comorbid neck and back pain (p < 0.001).

### YSR Syndrome Scores and sex differences

Descriptive statistics for the eight YSR syndrome scales are reported in Table 1, overall and by sex. Females displayed significantly higher mean Somatic, Anxious/Depressed, Withdrawn, Aggressive, Thought and Attention problem scores (p < 0.001 to 0.035), whilst males displayed significantly higher mean Rule-breaking scores (p = 0.013), and sexes had comparable mean Social scores (p = 0.125).

**Table 1:** Pain prevalence and YSR syndrome scale scores, overall and by gender

	Males n = 806	Females n = 774	p-value		Overall n = 1580
**Spinal Pain: (n, %)**					
No Pain	482 (59.8)	404 (52.2)			886 (56.1)
Neck pain only	111 (13.8)	134 (17.3)			245 (15.5)
Back pain only	140 (17.3)	100 (12.9)			240 (15.2)
Neck and back pain	73 (9.1)	136 (17.6)	< 0.001		209 (13.2)
**YSR Syndrome Scores:mean (sd)**					
Somatic (0-18)	2.7 (2.5)	3.4 (2.7)	< 0.001		3.1 (2.6)
Anxious/Depressed (0-32)	3.3 (3.4)	5.1 (4.7)	< 0.001		4.2 (4.2)
Withdrawn (0-14)	2.5 (1.9)	3.2 (2.2)	< 0.001		2.9 (2.1)
Aggressive (0-38)	8.0 (5.1)	8.5 (5.1)	0.035		8.3 (5.1)
Rule-Breaking (0-22)	3.1 (2.3)	2.8 (2.5)	0.013		2.9 (2.4)
Social (0-16)	2.6 (2.1)	2.8 (2.2)	0.125		2.7 (2.1)
Thought (0-14)	2.6 (2.1)	2.9 (2.4)	0.035		2.8 (2.2)
Attention (0-18)	4.8 (3.0)	5.3 (3.1)	0.001		5.0 (3.1)

### Association of YSR syndrome scale scores with monthly prevalence of spinal pain

Associations between spinal pain and YSR syndrome scores, as evaluated by multinomial logistic regression, are displayed in Table 2. Separate estimates are presented for males and females in the presence of a significant sex interaction term.

Adolescents with higher Somatic, Anxious/Depressed and Withdrawn (components of Internalising Disorders) were at greater risk of experiencing neck pain or back pain in isolation. Furthermore, adolescents with higher scores on these scales were at even greater risk of experiencing comorbid back and neck pain, with the risk of comorbid pain being significantly greater than neck or back pain alone. Although in general risk ratios were higher in females than males, the only significant sex interaction term was for the contrast between back pain only and no pain for the Withdrawn scale score, where the effect for females was significantly greater than that for males (p = 0.069).

Similar results were found for the Aggressive and Rule-Breaking scale scores (components of Externalising Disorders). Adolescents with higher Aggression scores were at greater risk of experiencing neck pain or back pain in isolation, and those with higher Rule-breaking scores at greater risk of experiencing back pain in isolation. For both scales higher scores were associated with an even greater risk of experiencing comorbid back and neck pain, with the risk of comorbid pain again being significantly greater than neck or back pain alone. No sex interactions were detected for these scales.

Higher Social scale scores were associated with greater risk for all three pain categories, but risk for comorbid pain was not significantly greater than neck or back pain in isolation. Higher Thought scale scores were associated with greater risk for all three pain categories, and the risk for comorbid pain was significantly greater than for back pain in isolation, but not neck pain. The effect of higher Thought scale scores for females was significantly greater than that for males (p = 0.052). Higher Attention scale scores were associated with greater risk for all three pain categories, and the risk for comorbid pain was significantly greater than for back pain or neck pain in isolation.

**Table 2:** Multinomial logistic regression results: Relative Risk Ratios for Back and/or Neck pain

		Neck pain only (16%)			Back pain only (15%)			Neck AND Back Pain (13%)		
		MOR	95%CI	p-value	MOR	95%CI	p-value	MOR	95%CI	p-value
**Somatic**	**Overall**	1.73	1.46-2.05	< 0.001	1.41	1.18-1.69	< 0.001	2.44^1,2^	2.05-2.90	< 0.001
										
**Anx/Dep**	**Overall**	1.43	1.20-1.70	< 0.001	1.38	1.15-1.66	0.001	1.94^1,2^	1.64-2.30	< 0.001
										
**WithDrawn**	**Overall**	1.52	1.23-1.88	< 0.001	1.51^3^	1.22-1.88	< 0.001	2.25^1,2^	1.82-2.79	< 0.001
	**Males**				1.25	0.92-1.69	0.152			
	**Females**				1.86	1.37-2.53	< 0.001			
										
**Aggressive**	**Overall**	1.39	1.14-1.69	0.001	1.49	1.22-1.1	< 0.001	1.91^1,2^	1.56-2.34	< 0.001
										
**Rule-break**	**Overall**	1.12	0.93-1.35	0.215	1.23	1.03-1.47	0.022	1.55^1,2^	1.31-1.85	< 0.001
										
**Social**	**Overall**	1.34	1.10-1.63	0.004	1.43	1.17-1.74	< 0.001	1.63	1.33-2.00	< 0.001
										
**Thought**	**Overall**	1.61	1.33-1.94	< 0.001	1.39^3^	1.14-1.70	0.001	1.98^1^	1.63-2.40	< 0.001
	**Males**				1.15	0.87-1.52	0.327			
	**Females**				1.71	1.29-2.26	< 0.001			
										
**Attention**	**Overall**	1.28	1.06-1.55	0.010	1.50	1.25-1.82	< 0.001	1.96^1,2^	1.61-2.38	< 0.001

(Reference group = NO neck OR back pain 56%). Multinomial Odds Ratios (MOR) are presented as ratio of risk for a change in the interquartile range of the score, i.e. moving from 25^th ^to 75^th ^percentile.

^1^Contrast to 'back pain only' also significant

^2^Contrast to 'neck pain only' also significant

^3^Gender interaction term p < 0.

## Discussion

This large study of over 1,500 Australian adolescents provides important prevalence data regarding the experience of spinal pain and mental health problems in this age group. Consistent with prevalence rates reported in other international studies [10] we found that nearly 44% of this sample of 14 year olds reported some kind of musculoskeletal spinal pain and approximately 13% had comorbid neck and back pain. This is a high figure considering that unlike other studies we did not include other pain categories such as abdominal pain or headache. In addition, we found a higher prevalence of neck pain and comorbid neck and back pain amongst females in this sample of adolescents. This finding is consistent with other studies that have reported an increased prevalence of pain in general amongst females [10,18]. However, our finding of a sex difference in the prevalence of musculoskeletal pain is in contrast to some other studies [19,20]. The point prevalence of mental health disorders in this cohort (based on YSR T scores of 60 or greater) was 18.0%, somewhat greater than others reported for adolescents in Australia and USA [1,2].

Our principal hypothesis that higher levels of psychological problems would be associated with increased odds of the experience of either back or neck pain, and further increased odds of co-morbid back and neck pain was supported. This finding occurred for all 8 of the syndrome scales making up the YSR. These associations were consistent across internalising and externalising scales and to a lesser extent across the third general scale, comprised of Social, Thought and Attention problems.

Our findings support those of Murphy and colleagues [14] who found that low back, upper back and neck pain were all associated with emotional problems. Importantly, our findings also support and extend those of Watson and colleagues [24] who found that the experience of low back pain was strongly related to a variety of emotional and behavioural problems in a similar sized cohort of 11-14 year olds. Watson and colleagues found associations between LBP and emotional, conduct and somatic complaints on the SDQ. The emotional and conduct scales of the SDQ closely approximate the internalising and externalising scales of the YSR used in this study. However, our findings extend those of Watson as we found relationships between emotional and behavioural problems and neck and back pain. Furthermore, the use of the YSR in this study has enabled more specific associations to be identified. For example, elevated scores on the Internalising/Emotional problems scale of the SDQ is made up of five items (often complains of headache; often worries; often unhappy/tearful; often nervous/clingy; often fearful). In contrast, the CBCL used in this study divides internalising problems into separate, more defined groupings, such as, Anxious/Depressed, Somatic Complaints and Withdrawn/Depressed. This is an important difference because in this study we were able to separate out specific associations between pain and certain components of internalising and externalising problems.

For example, within the Internalising scale of the CBCL, those adolescents with elevated scores on the Somatic Complaints scale had the highest risk of both neck and back pain. This provides important information as it suggests that the experience of comorbid spinal pain is associated with higher levels of general somatic focus (e.g. reporting eye problems, nausea, headaches). In this study, adolescents who reported elevated symptoms on the Withdrawn/Depressed scale were also at increased risk of comorbid back and neck pain. This scale is made up of items that most closely align to depression (e.g. won't talk, sad, enjoys little). This is an important association because unlike the SDQ, the CBCL separates somatic symptoms from withdrawn/depressed symptoms allowing a 'purer' measure of the unique associations between this syndrome and back and neck pain. The fact that this scale had particularly high associations with the experience of comorbid spinal pain in this sample has important potential implications for future research. Given the high incidence of depressive disorders in adolescence and the strong relationship to subsequent adult depression, this is an area for further consideration. It is also particularly noteworthy that the only significant sex interaction between pain location and psychological distress occurred on this scale. That is, females with higher scores on the Withdrawn/Depressed scale had a significantly greater risk of back pain.

We also found that adolescents with elevated scores on the Anxious/Depressed scale were at increased risk of comorbid back and neck pain. This scale includes symptoms such as 'fears school', 'nervous', 'worries' and thus captures the presence of anxiety symptoms. As with depressive disorders, anxiety disorders often have an onset in adolescence and are highly comorbid with depression. Also as predicted, we found significant associations between scores on the externalising scale (Rule-breaking and Aggressive Behaviour) and comorbid back and neck pain. This finding is also consistent with Watson and colleagues [24] and suggests that there is a strong association between behaviours such as breaking rules and acting out and the concurrent experience of back and neck pain.

The finding that both internalising and externalising symptoms are associated with comorbid back and neck pain warrants further consideration. The report for greater comorbidity between psychological factors and spinal pain in two locations is consistent with findings of psychological distress being greater in widespread pain disorders [40]. To our best knowledge this is the first study to report this finding in adolescents and there are a number of possible explanations.

The potential reciprocal causal relationship between spinal pain and psychological factors has created a dilemma [41]. On the one hand it is recognized that pain in adolescents can result in widespread negative effects on many aspects of life [42], which has the potential to negatively influence psychological factors. For example, Carroll and colleagues [28] found that spinal pain (neck or low back pain) was associated with new episodes of depression lending support to the view that the experience of pain predisposes an individual to later depression. On the other hand there is also evidence that musculoskeletal pain is preceded by psychological problems such as depression in adolescents [20,40] as well as in adults [28,43].

This potentially reciprocal relationship could be mediated via a number of different pathways. For example, it has been proposed that the relationship between depression and pain may be associated with pathophysiological disturbances related to serotonin metabolism [20] and pain sensitization [40]. Furthermore, higher levels of distress are associated with stress biomarkers which are known to be related to spinal pain [44] and muscle tension [45]. It has also been proposed that behaviours such as passivity and inactivity, which are known features of depression, may also magnify sensory input such as pain [28]. Previous research has reported that psychological factors are also known to be associated with the adoption of specific postures known to be associated with spinal pain, suggesting a biomechanical relationship may also exist [46,47]. It must also be considered that psychosocial factors, such as socioeconomic disadvantage, parental mental illness, family dysfunction, and stressful life-events are common in those who experience mental health problems and pain [6,7,24,40,48]. These psychosocial circumstances are known to cause disturbances in early psychoneuroendocrine and neurological development [49-51]. Whatever the direction of the relationship between pain and psychological factors, it has been proposed that they both potentially contribute to a vicious cycle perpetuating both states [41,52].

Being cross-sectional, the current study did not address the question of causality but found evidence for the presence of both pain and psychological problems amongst adolescents. Some authors [24,53] have argued that this pattern of findings can be best explained from a developmental perspective; adolescents may be learning how to express pain in general. This view suggests that emotional and physiological pain could be difficult for the adolescent to separate and thus they tend to positively endorse items from both areas.

Our second principal hypothesis concerning sex was also supported. Girls had significantly higher scores than boys on most of the scales of the CBCL (Somatic, Anxious/Depressed, Withdrawn, Aggressive, Thought and Attention). Our prediction that girls and boys would have similar associations between psychological distress and back/neck pain was partially supported. On the whole, the relationship between pain and psychological symptoms was stronger for girls, although the only significantly different association was on the Withdrawn/Depressed scale where girls had higher odds of back pain than boys.

As this study is cross-sectional it does not provide information as to the nature of the development of pain or psychological problems over time nor the direction of causality nor the influence of other factors on the pathways to spinal pain and psychological problems. A longitudinal exploration of these variables is essential given that there is theoretical support suggesting that adolescents who report pain and elevated psychological distress may be at increased risk of future difficulties. Our research group is currently investigating this question by following up this cohort of adolescents.

## Conclusion

This is the first large-scale study to show that the experience of mental health problems in adolescence is associated with increased risk for comorbid back and neck pain. The current findings highlight the importance of a comprehensive clinical assessment of adolescent functioning to include both physical symptoms (e.g. pain location, site, impairment) as well as psychological symptoms (mood and other psychosocial factors). Regardless of the question of causality, it is clear that the experience of physical and emotional pain is not independent and thus in practice, both aspects must be considered in order to obtain optimal client outcomes.

## Competing interests

The authors declare that they have no competing interests.

## Authors' contributions

CR drafted the manuscript. AS carried out the analysis and edited the manuscript. PO participated in the study design, coordination and manuscript writing. GK participated in the design of the study, study coordination and manuscript review. LS conceived of the study, and participated in its design and coordination and helped to draft the manuscript. All authors read and approved the final manuscript.

## Pre-publication history

The pre-publication history for this paper can be accessed here:

http://www.biomedcentral.com/1471-2458/11/382/prepub
